# Higher cardiovascular risk observed with beta-blockers in CKD patients without prior cardiovascular disease

**DOI:** 10.1093/ckj/sfag204

**Published:** 2026-06-12

**Authors:** Seung Hyun Han, Mina Kim, Jungkuk Lee, Sang Youb Han

**Affiliations:** Department of Internal Medicine, Division of Nephrology, Inje University Ilsan Paik Hospital, Goyang, Gyunggi, South Korea; Department of Data Science, Hanmi Pharm. Co., Ltd, Seoul, South Korea; Department of Data Science, Hanmi Pharm. Co., Ltd, Seoul, South Korea; Department of Internal Medicine, Division of Nephrology, Inje University Ilsan Paik Hospital, Goyang, Gyunggi, South Korea

**Keywords:** beta-blockers, chronic kidney disease, major cardiovascular events, mortality

## Abstract

**Background:**

Chronic kidney disease (CKD) is strongly linked to cardiovascular disease (CVD) risk. Although beta-blockers (BBs) are widely used for CVD prevention, their effectiveness and safety in CKD patients without established CVD remain uncertain.

**Methods:**

We conducted a nationwide cohort study using Korean health insurance data. CKD patients without established CVD between 2012 and 2015 were identified based on an estimated glomerular filtration rate (eGFR) < 60 ml/min/1.73 m^2^ on at least two occasions. BB users were defined as those prescribed BBs for ≥6 months. Primary outcomes were all-cause mortality and 4-point major adverse cardiovascular events (MACE), including cardiovascular death, non-fatal myocardial infarction, stroke, and hospitalization for heart failure. A 1:2 propensity score matching was performed, and Cox proportional hazards models were applied. Subgroup analyses were also conducted by eGFR category and BB agents.

**Results:**

After matching, 9067 BB users and 17 094 non-users were included. BB use was associated with increased risks of all-cause mortality (hazard ratio [HR], 2.09; 95% confidence interval [CI], 1.96–2.24) and MACE (HR, 1.33; 95% CI, 1.26–1.41). The increased risks were consistent across individual outcomes, including cardiovascular death (HR, 2.07; 95% CI, 1.66–2.57), myocardial infarction (HR, 1.29; 95% CI, 1.13–1.46), stroke (HR, 1.21; 95% CI 1.13–1.31), and heart failure hospitalization (HR, 1.58; 95% CI, 1.44–1.72). Subgroup analysis showed greater mortality risk at lower eGFR levels and a higher risk of adverse events with carvedilol compared with other BBs.

**Conclusions:**

Among CKD patients without established CVD, BB use was associated with increased mortality and cardiovascular risk. Further studies are needed to clarify whether these associations reflect treatment effects or underlying patient risk.

KEY LEARNING POINTS **What was known:**

- Chronic kidney disease (CKD) is associated with markedly increased cardiovascular risk, and beta-blockers are widely prescribed for blood pressure control or presumed cardioprotective benefit.
- Robust evidence supporting beta-blocker use for primary cardiovascular prevention in CKD patients without established cardiovascular disease has been limited and inconsistent.

**This study adds:**

- Beta-blocker use was associated with increased risks of all-cause mortality and major adverse cardiovascular events, with the most pronounced increase observed for cardiovascular death and heart failure hospitalization.
- The associations were consistent across CKD stages and beta-blocker subclasses, suggesting that the cardiovascular benefits observed in other populations may not directly extend to CKD patients without established cardiovascular disease.

**Potential impact:**

- These findings highlight a clinically important discrepancy between prescribing patterns and actual patient risk, underscoring the need for more careful evaluation of beta-blocker use in CKD patients without established cardiovascular disease.
- Adoption of more individualized antihypertensive strategies may help optimize cardiovascular outcomes in this vulnerable population.

## INTRODUCTION

Chronic kidney disease (CKD) affects over 10% of the global population and is strongly associated with increased cardiovascular disease (CVD) and premature mortality [1, 2]. As kidney function declines, the prevalence of CVD, including coronary artery disease (CAD), heart failure (HF), and arrhythmias increases substantially. In CKD stages 4–5, ∼50% of patients are affected, and 40%–50% of deaths are due to cardiovascular causes, nearly twice that observed in individuals with preserved kidney function [3, 4]. Despite advances in kidney and cardiovascular care, the excess cardiovascular burden in CKD remains a major clinical challenge.

Beta-blockers (BBs) are widely used for the management of HF with reduced ejection fraction, CAD, and certain arrhythmias, and have been shown to reduce mortality and secondary cardiovascular events [5–8]. Clinical guidelines recommend BBs for patients with established CVD, particularly those with systolic HF or recent myocardial infarction (MI) [9, 10]. In CKD, sympathetic hyperactivity, vascular remodeling, and elevated cardiovascular risk [4, 11, 12] provide a pathophysiologic rationale for BB therapy. Consequently, BBs are commonly considered in this population.

However, the role of BBs in CKD remains uncertain. Earlier trials evaluating BBs as primary antihypertensive agents demonstrated inferior outcomes compared with renin–angiotensin system inhibitor (RASi) or calcium channel blockers (CCBs) [13, 14], whereas more recent studies have suggested potential cardiovascular benefits in CKD population [15–17]. Nonetheless, concerns such as bradycardia or hypotension have limited their widespread use in clinical practice [18, 19], and evidence in advanced CKD or dialysis populations remains scarce due to their underrepresentation in pivotal cardiovascular trials [20, 21]. In addition, pharmacologic heterogeneity among BB subclasses may contribute to inconsistent findings, as vasodilatory BBs such as carvedilol and nebivolol differ in their cardiovascular and hemodynamic effects from traditional agents like atenolol [22, 23].

Given this therapeutic uncertainty, further large-scale, real-world investigations are needed to clarify the benefit–risk profile of BB therapy across the CKD spectrum. Using a large nationwide Korean cohort, we evaluated the association between BB use and long-term clinical outcomes, including all-cause mortality and major adverse cardiovascular events (MACE), to provide additional real-world evidence that may guide individualized cardiovascular risk management in CKD.

## MATERIALS AND METHODS

### Data source

The study is a retrospective cohort based on claim data from the Korean National Health Insurance Service (NHIS) database. The NHIS database is a comprehensive nationwide health insurance claims system covering 97% of the Korean population since 2002, making it the largest and representative sources for epidemiological and clinical research in Korea. The database provides extensive healthcare detailed data, including claimed medical costs, prescribed medications, procedures, laboratory results, and diagnosis coded according to the International Classification of Diseases, 10th Revision (ICD-10). Detailed descriptions of the NHIS dataset have been documented in previous publication [24]. The study was approved by the Institutional Review Board of Inje University Ilsan Paik Hospital (IRB No. 2020-06-034-009).

### Study design and population

**Figure 1:**
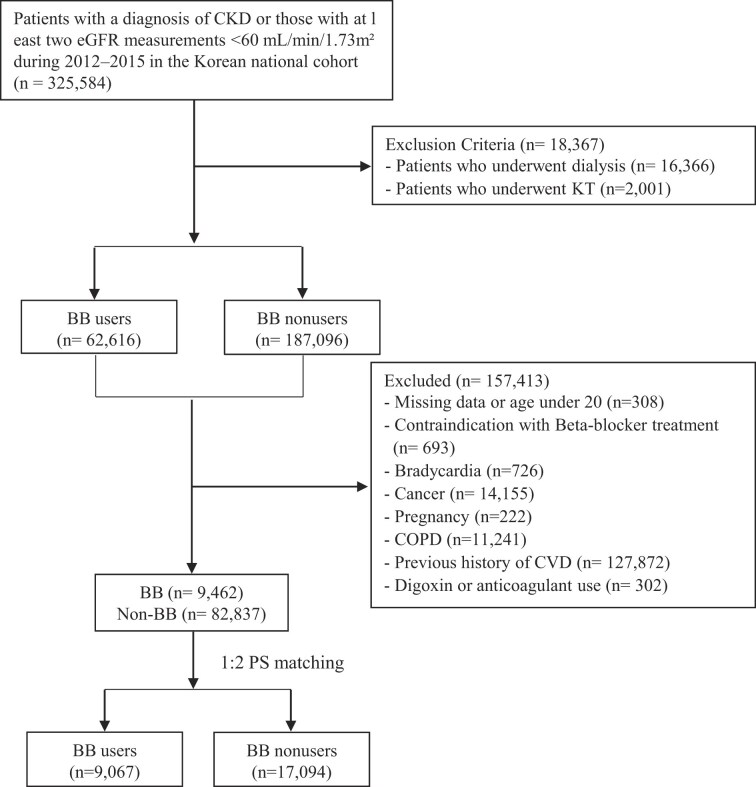
Study flow chart. Abbreviations: CKD, chronic kidney disease; eGFR, estimated glomerular filtration rate; BB, beta-blocker; COPD, chronic obstructive pulmonary disease.

A total of 325 584 patients who were diagnosed with CKD were identified. CKD was defined by the presence of a diagnostic code for CKD (ICD-10 codes, N18.x) or by at least two eGFR measurements <60 ml/min/1.73 m² obtained between 2012 and 2015. As the NHIS health check-up is conducted biennially, repeated eGFR measurements were separated by at least 1–2 years. Subsequently, patients who had already undergone dialysis (*n* = 16 366) or kidney transplantation (*n* = 2001) during the baseline period were excluded (Fig. 1).

We further excluded individuals with missing data, those aged <20 years, and those with contraindications to BB therapy, including bradycardia or the presence of implanted cardiac devices such as pacemakers, cardiac resynchronization therapy systems, and implantable cardioverter–defibrillators (ICDs). Patients with malignancy, pregnancy, or chronic obstructive pulmonary disease were also excluded. To restrict the cohort to individuals at risk of incident cardiovascular events, those with a prior history of CVD—including MI, coronary artery disease with revascularization, congestive HF, stroke or transient ischemic attack, and arrhythmias—were excluded. Furthermore, patients receiving digoxin or anticoagulants (warfarin or oral anticoagulants) were excluded to minimize confounding by underlying cardiovascular conditions. A detailed list of diagnostic and procedure codes used for these definitions is provided in Supplementary Table S1.

### Exposure

BB exposure was identified using prescription records from the Korean Drug and Anatomical Therapeutic Chemical Codes. For BB users, the index date was defined as the date of the first BB prescription after CKD diagnosis. Patients were classified as BB users who received BB prescriptions for at least six consecutive months. A prescription gap of more than 6 months was regarded as treatment discontinuation. Non-BB users were identified as those without BB prescriptions within a prespecified observation window after CKD diagnosis. An index date was assigned within the same window to align follow-up between groups. The length of the observation window was set at 4 years to allow sufficient time for exposure classification.

To minimize baseline imbalance, propensity score (PS) matching was then performed at 1:2 ratio. PSs were estimated using logistic regression including baseline demographic characteristics (age, sex), clinical factors (eGFR, systolic and diastolic blood pressure, body mass index [BMI], and albuminuria), comorbidities (hypertension, diabetes, dyslipidemia, and peripheral arterial disease), and concomitant medications. Medications assessed in 180 days before the index date which included RASi, CCB, statins, diuretics, and antiplatelet agents. Covariate balance was evaluated using absolute standardized differences (ASD), with ASD < 0.10 considered acceptable. The final matched cohort included 9067 BB users and 17 094 non-users with well-balanced baseline characteristics.

### Outcomes

The primary outcomes were all-cause mortality and 4-point MACE, defined as a composite of cardiovascular (CV) death, non-fatal MI, ischemic stroke, and hospitalization for HF. CV death was defined as death in which a CVD was recorded as the underlying cause of death on death certificates collected by Statistics Korea, under the Ministry of Economy and Finance of South Korea (ICD-10 codes I20–I25 and I60–I64). Non-fatal events were ascertained using ICD-10 codes: MI (I21–I23), ischemic stroke (I63, I64), and HF (I50). Participants were followed from the index date until the first occurrence of any outcome, or the end of the study period (31 December 2022). To allow sufficient pharmacologic induction, the primary analysis considered events occurring after day 180 from the index date [25].

### Statistical analysis

Baseline characteristics including demographics, comorbidities, and medications were summarized as frequencies (%) for categorical variables and as means with standard deviations (SDs) for continuous variables. To minimize baseline imbalances and confounding, PS matching was conducted at a 1:2 ratio using a hierarchical sequence with a caliper width of 0.02 [26].

The association between beta-blocker use and outcomes was evaluated using Cox proportional hazards models to estimate hazard ratios (HRs) with 95% confidence intervals (CIs). Both crude and multivariable-adjusted models were constructed. The multivariable models adjusted for a comprehensive set of covariates. Demographic variables included age, sex, location (urban or rural), and household income level (categorized into quintiles of national health insurance premium). Smoking status (never, former, or current) and alcohol consumption (frequency per week and average amount per occasion) were also included. Clinical measures included eGFR, BMI, hemoglobin, and urine albuminuria (negative, trace, or ≥1+ by dipstick test). Comorbidities included hypertension, diabetes, dyslipidemia, and peripheral arterial disease. Medication use included RASi, CCB, statins, diuretics, and antiplatelet agents. In addition, to account for changes in hypertension severity during follow-up, the number of antihypertensive drug classes was included as a time-varying covariate.

Survival probabilities and cumulative incidence curves were generated using the Kaplan–Meier method, and differences between groups were assessed with log-rank tests. Subgroup analyses were performed according to age, sex, diabetes status, blood pressure control, and baseline kidney function. In addition, to address potential immortal time bias, a 1-year landmark sensitivity analysis was performed. BB exposure was reclassified based on prescription status at 1 year after CKD diagnosis, and patients who experienced the outcomes within 1 year excluded.

A two-tailed *P*-value <0.05 was considered statistically significant. All statistical analyses were conducted using SAS Enterprise Guide software version 9.4 (SAS Institute Inc, Cary, North Carolina, USA).

## RESULTS

A total of 92 601 patients with CKD were initially identified, of whom 9462 were prescribed BBs and 82 837 were not (Table 1). Before matching, BB users were older (64.1 ± 11.1 years), more often male (56.7%), and had a higher prevalence of hypertension (99.0%), diabetes (55.1%), and dyslipidemia (76.8%). They also had slightly higher baseline systolic (134.0 ± 17.2 mmHg) and diastolic (80.5 ± 11.4 mmHg) blood pressures compared with non-BB users. Among BB users, 32.6% received carvedilol and 8.0% received nebivolol. After 1:2 PS matching, 9067 BB users and 17 094 non-users were included. Baseline demographic, clinical, and medication characteristics were well balanced between the two groups, with all ASDs < 0.1, except for location. The distributions of PS were nearly identical, indicating successful matching (Table 1).

**Table 1:** Baseline characteristics.

Characteristic	Full cohort			1:2 PS matched cohort		
	BB user (*N* = 9462)	Non-BB user (*N* = 82 837)	ASD	BB user (*N* = 9067)	Non-BB user (*N* = 17 094)	ASD
Age, years	64.1 (11.1)	60.2 (11.9)	0.3339	64.3 (10.9)	64.5 (10.7)	0.0134
Male, *n* (%)	5367 (56.7)	41 288 (49.8)	0.1319	5105 (56.3)	9541 (55.8)	0.0098
Location, rural (%)	1204 (12.7)	11 291 (13.6)	0.0293	1149 (12.7)	2644 (15.5)	0.1551
Household income level, *n* (%)			0.0553			0.0482
Medical aid (lowest)	34 (0.4)	329 (0.4)		28 (0.3)	57 (0.3)	
1st–5th percentile	110 (1.2)	935 (1.1)		106 (1.2)	199 (1.2)	
6th–10th percentile	484 (5.1)	3379 (4.1)		464 (5.1)	711 (4.2)	
11th–15th percentile	1566 (16.6)	12 715 (15.3)		1491 (16.4)	2640 (15.4)	
16th–20th percentile	7268 (76.8)	65 479 (79.0)		6978 (77.0)	13 487 (78.9)	
Smoking, *n* (%)			0.0803			0.0318
Never	5730 (60.6)	54 616 (65.9)		5665 (62.5)	10 910 (63.8)	
Former	1963 (20.7)	15 059 (18.2)		1920 (21.2)	3620 (21.2)	
Current	1511 (16.0)	12 528 (15.1)		1475 (16.3)	2562 (15.0)	
Alcohol consumption						
Frequency (/week)	0.9 (1.5)	0.7 (1.3)	0.0968	0.9 (1.5)	0.8 (1.5)	0.0403
Amount (glass)	4.0 (4.1)	3.6 (4.0)	0.1033	4.0 (4.1)	3.7 (4.1)	0.0504
Clinical measures						
BMI (kg/m^2^),	25.5 (3.4)	24.3 (3.1)	0.3785	25.4 (3.3)	25.3 (3.2)	0.0357
SBP (mmHg),	134.0 (17.2)	125.9 (15.2)	0.5023	133.6 (16.8)	133.2 (16.0)	0.0265
DBP (mmHg),	80.5 (11.4)	77.4 (10.0)	0.2835	80.3 (11.1)	80.1 (10.5)	0.0183
CKD stage			0.3016			0.029
40–60 (3b)	7470 (78.9)	74 317 (89.7)		7174 (79.1)	13 716 (80.2)	
30–45 (3a)	1650 (17.4)	7544 (9.1)		1604 (17.7)	2908 (17.0)	
15–30 (4)	293 (3.1)	693 (0.8)		249 (2.7)	389 (2.3)	
<15 (5)	49 (0.5)	283 (0.3)		40 (0.4)	81 (0.5)	
eGFR (ml/min/1.73 m^2^),	49.7 (9.4)	52.6 (7.3)	0.352	49.9 (9.2)	50.4 (8.6)	0.0598
Hb (g/dl)	13.4 (1.8)	13.6 (1.7)	0.11	13.4 (1.8)	13.5 (1.7)	0.0163
FBS (mg/dl)	111.8 (34.2)	103.6 (28.9)	0.2587	111.6 (34.0)	111.0 (35.0)	0.017
Urine albuminuria, *n* (%)			0.2969			0.0385
Negative	7136.0 (75.4)	71 858.0 (86.7)		7072 (78.0)	13 591 (79.5)	
+/–	374 (4.0)	2645 (3.2)		368 (4.1)	701 (4.1)	
≥+1	1952 (20.6)	8334 (10.1)		1627 (17.9)	2802 (16.4)	
Comorbidities history, *n* (%)						
Hypertension	9366 (99)	41 155 (49.7)	1.3673	8980 (99.0)	16 924 (99.0)	0.0036
Diabetes	5210 (55.1)	29 183 (35.2)	0.4067	4942 (54.5)	9235 (54.0)	0.0096
Dyslipidemia	7269 (76.8)	44 138 (53.3)	0.5095	6917 (76.3)	12 959 (75.8)	0.0112
Peripheral artery disease	3462 (36.6)	18 641 (22.5)	0.3125	3312 (36.5)	6154 (36.0)	0.011
Medication, *n* (%)						
Carvedilol	3086 (32.6)	0(0)	0.9839	2890 (31.9)	0 (0)	0.9673
Nebivolol	760 (8.0)	0(0)	0.4179	700 (7.7)	0 (0)	0.4091
Other beta-blocker	5940 (62.8)	0(0)	1.8366	5796 (63.9)	0 (0)	1.8825
RAS inhibitors	5837 (61.7)	28 230 (34.1)	0.5751	5538 (61.1)	10 582 (61.9)	0.017
Calcium channel blockers	5297 (56.0)	14 627 (17.7)	0.8659	5005 (55.2)	9230 (54.0)	0.0242
Diuretics	2894 (30.6)	6401 (7.7)	0.607	2672 (29.5)	4381 (25.6)	0.086
Statins	5193 (54.9)	25 262 (30.5)	0.5087	4925 (54.3)	9163 (53.6)	0.0143
Antiplatelet agents	3208 (33.9)	11 226 (13.6)	0.4927	3069 (33.8)	5661 (33.1)	0.0155

Data are presented as mean (SD) for continuous variables and number (%) for categorical variables.

Abbreviations: ASD, absolute standardized difference; BB, beta-blocker; BMI, body mass index; SBP, systolic blood pressure; DBP, diastolic blood pressure; CKD, chronic kidney disease; eGFR, estimated glomerular filtration rate; Hb, hemoglobin; FBS, fasting blood sugar; RAS, renin–angiotensin system.

### Main outcomes

During a median follow-up of 8.1 years (IQR, 4.6–10.2) in the full cohort, a total of 8560 deaths and 14 267 MACE were recorded (Table 2). In the matched cohort, the median follow-up was 7.9 years (IQR, 4.5–10.0), during which 1435 deaths and 2420 MACE occurred.

**Table 2:** Beta-blocker use and risks of mortality and major cardiovascular events in the full and matched cohorts.

Main outcome			HR (95% CI)		
	No. of events BB/non-BB	Incidence rate	Full cohort, crude	Full cohort, adjusted	PSM cohort, adjusted
All-cause mortality	1411/7149	16.3/8.9	1.89 (1.79–2.00)	2.13 (2.00–2.27)	2.09 (1.96–2.24)
MACE	2386/11 881	30.5/15.7	2.01 (1.92–2.10)	1.30 (1.24–1.37)	1.33 (1.26–1.41)
MACE components					
CV death	152/646	1.8/0.8	2.24 (1.88–2.67)	2.25 (1.85–2.74)	2.07 (1.66–2.57)
Non-fatal MI	468/2211	5.5/2.8	2.02 (1.83–2.24)	1.30 (1.16–1.46)	1.29 (1.13–1.46)
Ischemic stroke	1374/7390	16.9/9.6	1.79 (1.69–1.89)	1.20 (1.13–1.28)	1.21 (1.13–1.31)
HF hospitalization	1056/3923	12.6/5.0	2.67 (2.49–2.85)	1.47 (1.36–1.59)	1.58 (1.44–1.72)

Median follow-up was 8.1 (IQR, 4.6–10.2) years in the full cohort and 7.9 (IQR, 4.5–10.0) years in the PSM cohort.

Incidence rates are per 1000 person-years.

Adjusted: age, sex, eGFR, smoking, alcohol, location, family income level, Hb, BMI, albuminuria, HTN, DM, DLP, PAD, RASi, CCB, diuretics, statins, antiplatelet agents, and number of hypertensive medications.

Abbreviations: BB, beta-blocker; HR, hazard ratio; CI, confidence interval; PSM, propensity-score matched; MACE, major adverse cardiovascular events; CV, cardiovascular; MI, myocardial infarction; HF, heart failure.

In the PSM analysis, BB users were associated with higher risks of both all-cause mortality (adjusted HR, 2.09; 95% CI, 1.96–2.24) and 4-point MACE (adjusted HR, 1.33; 95% CI, 1.26–1.41) compared with non-BB users. These associations were consistent in the full cohort, both in crude and adjusted analyses (HR, 2.13; 95% CI, 2.00–2.27 for mortality; HR, 1.30; 95% CI, 1.24–1.37 for MACE).

Analysis of individual MACE components in the matched cohort showed consistently higher risks among BB users, with the most pronounced increase observed for CV death (HR, 2.07; 95% CI, 1.66–2.57) and HF hospitalization (HR, 1.58; 95% CI, 1.44–1.72). Non-fatal MI (HR, 1.29; 95% CI, 1.13–1.46) and ischemic stroke (HR, 1.21; 95% CI, 1.13–1.31) also showed elevated risks.

**Figure 2:**
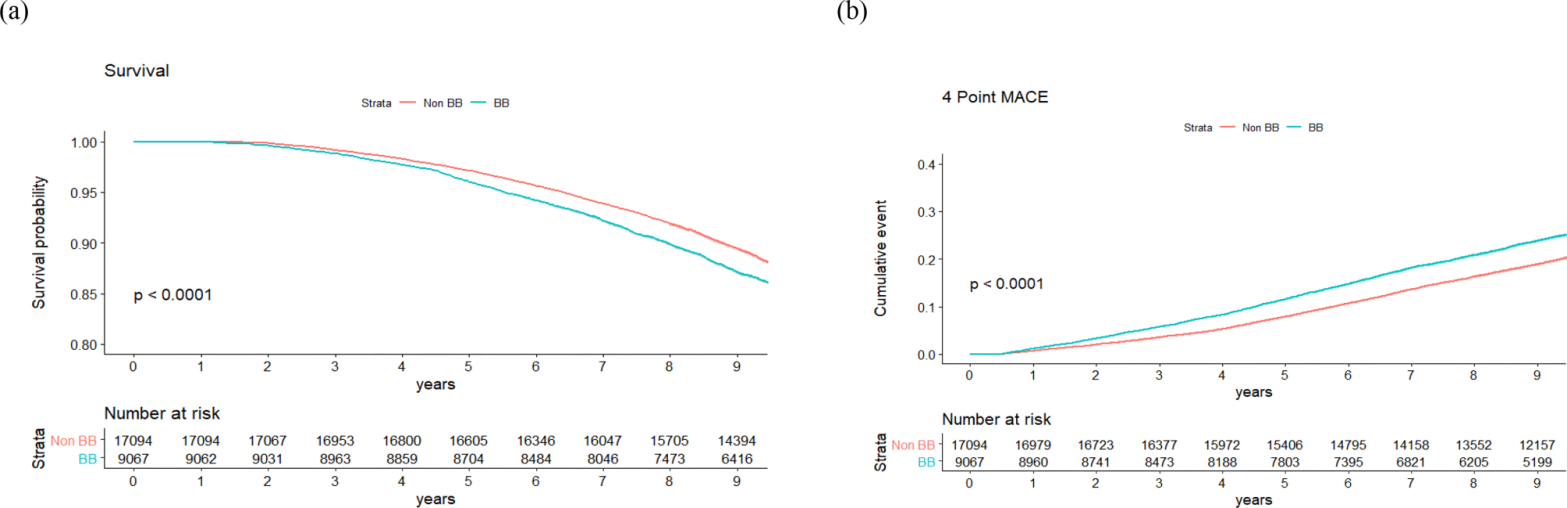
Kaplan–Meier curves for (a) all-cause mortality and (b) major cardiovascular events in the matched cohort.

Kaplan–Meier analysis also demonstrated lower survival and higher cumulative incidence of MACE among BB users in the matched cohort (all *P* < 0.05) (Fig. 2). Similar trends were observed for each MACE component, whereas CV death showed a weaker separation, with borderline significance (*P* = 0.05) (Fig. S1).

These patterns were consistent in the full cohort, where BB use remained associated with higher risks of CV death, MI, stroke and HF hospitalization, in both crude and adjusted analysis.

### Subgroup analysis by kidney function

When stratified by baseline kidney function (Table 3), the association between BB use and adverse outcomes was consistently elevated across all eGFR categories, except in patients with eGFR < 15 ml/min/1.73 m². In the matched cohort, BB use was associated with higher risks of all-cause mortality across declining eGFR categories, with the strongest associations observed in patients with eGFR 30–45 ml/min/1.73 m² (HR, 2.28; 95% CI, 1.98–2.61). In contrast, the excess risk of MACE showed a progressive increase with declining kidney function, with the highest estimate observed in advanced CKD, in patients with eGFR 15–30 ml/min/1.73 m² (HR, 1.43; 95% CI, 1.03–1.99). Analyses of individual MACE components revealed that the risks of CV death (HR, 2.14; 95% CI, 1.65–2.78) and HF hospitalization (HR, 1.60; 95% CI, 1.44–1.78) were most pronounced in patients with eGFR 45–60 ml/min/1.73 m², while ischemic stroke was most evident in those with eGFR 30–45 ml/min/1.73 m² (HR, 1.39; 95% CI, 1.18–1.63). The excess risk of non-fatal MI was particularly pronounced in patients with eGFR 15–30 ml/min/1.73 m² (HR, 2.38; 95% CI, 1.19–4.76).

**Table 3:** Adjusted HRs for the all-cause mortality and MACE according to the baseline kidney function.

Main outcome	HR (95% CI)		
	Full cohort, crude	Full cohort, adjusted	PSM cohort, adjusted
All-cause mortality			
45 ≤ eGFR < 60	1.83 (1.71–1.96)	2.09 (1.94–2.25)	2.05 (1.89–2.23)
30 ≤ eGFR < 45	1.44 (1.28–1.62)	2.25 (1.98–2.56)	2.28 (1.98–2.61)
15 ≤ eGFR < 30	1.29 (0.97–1.70)	1.83 (1.32–2.54)	1.72 (1.21–2.43)
eGFR < 15	4.82 (2.16–10.78)	3.03 (0.50–18.30)	NA
MACE			
45 ≤ eGFR < 60	1.99 (1.89–2.09)	1.29 (1.22–1.36)	1.32 (1.23–1.41)
30 ≤ eGFR < 45	1.57 (1.42–1.74)	1.34 (1.19–1.50)	1.37 (1.21–1.55)
15 ≤ eGFR < 30	1.57 (1.22–2.01)	1.45 (1.07–1.97)	1.43 (1.03–1.99)
eGFR < 15	3.66 (1.94–6.92)	0.86 (0.29–2.58)	0.30 (0.06–1.66)
MACE components			
CV death			
45 ≤ eGFR < 60	2.36 (1.92–2.90)	2.36 (1.88–2.98)	2.14 (1.65–2.78)
30 ≤ eGFR < 45	1.17 (0.79–1.74)	1.94 (1.26–2.98)	1.82 (1.15–2.90)
15 ≤ eGFR < 30	1.71 (0.75–3.93)	2.08 (0.75–5.74)	2.42 (0.81–7.22)
eGFR < 15	12.15 (1.10–134.00)	NA	NA
Non-fatal MI			
45 ≤ eGFR < 60	1.97 (1.76–2.21)	1.27 (1.12–1.45)	1.25 (1.08–1.45)
30 ≤ eGFR < 45	1.63 (1.28–2.08)	1.23 (0.93–1.61)	1.19 (0.88–1.60)
15 ≤ eGFR < 30	2.80 (1.68–4.67)	2.17 (1.19–3.97)	2.38 (1.19–4.76)
eGFR < 15	1.71 (0.20–14.69)	NA	NA
Ischemic stroke			
45 ≤ eGFR < 60	1.77 (1.65–1.89)	1.17 (1.08–1.26)	1.18 (1.08–1.28)
30 ≤ eGFR < 45	1.53 (1.34–1.74)	1.36 (1.17–1.58)	1.39 (1.18–1.63)
15 ≤ eGFR < 30	1.11 (0.77–1.61)	1.09 (0.69–1.71)	0.94 (0.57–1.55)
eGFR < 15	3.17 (1.38–7.29)	0.77 (0.17–3.59)	0.13 (0.01–1.75)
HF hospitalization			
45 ≤ eGFR < 60	2.62 (2.42–2.84)	1.47 (1.34–1.61)	1.60 (1.44–1.78)
30 ≤ eGFR < 45	1.90 (1.64–2.20)	1.44 (1.22–1.70)	1.51 (1.26–1.81)
15 ≤ eGFR < 30	1.94 (1.38–2.73)	1.53 (1.01–2.31)	1.55 (0.99–2.44)
eGFR < 15	5.84 (2.45–13.92)	1.17 (0.22–6.18)	0.18 (0.00–17.76)

Adjusted: age, sex, eGFR, smoking, alcohol, location, family income level, Hb, BMI, albuminuria, HTN, DM, DLP, PAD, RASi, CCB, diuretics, statins, antiplatelet agents, and number of hypertensive medications.

Abbreviations: HR, hazard ratio; CI, confidence interval; PSM, propensity-score matched; MACE, major adverse cardiovascular events; CV, cardiovascular; MI, myocardial infarction; HF, heart failure.

### Comparative outcomes among beta-blockers

Given the pharmacologic diversity among beta-blockers, we conducted additional analyses to examine whether clinical outcomes varied according to the specific agent prescribed (Table 4). In the full and matched cohorts, all BB subclasses were associated with increased risks of both all-cause mortality and MACE. Carvedilol was associated with increased risks of mortality (HR, 2.22; 95% CI, 1.99–2.48) and MACE (HR, 1.43; 95% CI, 1.31–1.55) in matched cohort. Nebivolol demonstrated similarly elevated risks of both mortality (HR, 2.00; 95% CI, 1.59–2.51) and MACE (HR, 1.38; 95% CI, 1.17–1.63), consistent with other BBs (For mortality, HR, 2.05; 95% CI, 1.89–2.22; for MACE, HR, 1.28; 95% CI, 1.20–1.37). In the analysis of individual MACE component, all BB subclasses were consistently associated with higher risks of CV death and HF hospitalization in the matched cohort. For ischemic stroke and non-fatal MI, carvedilol and other BBs showed associations, whereas nebivolol did not reach statistical significance (stroke: HR, 1.23; 95% CI, 0.99–1.54; MI: HR, 1.15; 95% CI, 0.78–1.70).

**Table 4:** Outcomes associated with the initiation of carvedilol, nebivolol, and other BBs in patients among CKD.

Main outcome	HR (95% CI)		
	Full cohort, crude	Full cohort, adjusted	PSM cohort, adjusted
All-cause mortality			
Carvedilol (*n* = 3001)	1.95 (1.77–2.14)	2.32 (2.10–2.57)	2.22 (1.99–2.48)
Nebivolol (*n* = 706)	1.70 (1.38–2.10)	2.14 (1.72–2.65)	2.00 (1.59–2.51)
Other BBs (*n* = 5850)	1.89 (1.76–2.03)	2.05 (1.90–2.20)	2.05 (1.89–2.22)
MACE			
Carvedilol	2.08 (1.93–2.24)	1.43 (1.33–1.55)	1.43 (1.31–1.55)
Nebivolol	2.08 (1.79–2.42)	1.45 (1.25–1.69)	1.38 (1.17–1.63)
Other BBs	1.97 (1.86–2.08)	1.23 (1.16–1.31)	1.28 (1.20–1.37)
MACE components			
CV death			
Carvedilol	2.31 (1.72–3.10)	2.39 (1.75–3.26)	2.11 (1.49–2.99)
Nebivolol	2.11 (1.13–3.94)	2.33 (1.23–4.41)	2.24 (1.13–4.42)
Other BBs	2.22 (1.79–2.76)	2.18 (1.72–2.75)	2.03 (1.57–2.62)
Non-fatal MI			
Carvedilol	2.13 (1.81–2.51)	1.34 (1.13–1.60)	1.32 (1.09–1.60)
Nebivolol	2.28 (1.65–3.16)	1.46 (1.05–2.04)	1.15 (0.78–1.70)
Other BBs	1.94 (1.72–2.20)	1.26 (1.10–1.44)	1.28 (1.10–1.49)
Ischemic stroke			
Carvedilol	1.78 (1.61–1.96)	1.32 (1.19–1.46)	1.30 (1.16–1.45)
Nebivolol	1.74 (1.42–2.13)	1.30 (1.06–1.60)	1.23 (0.99–1.54)
Other BBs	1.80 (1.68–1.93)	1.14 (1.06–1.23)	1.17 (1.08–1.28)
HF hospitalization			
Carvedilol	2.89 (2.59–3.22)	1.59 (1.42–1.79)	1.67 (1.47–1.90)
Nebivolol	3.03 (2.44–3.76)	1.72 (1.37–2.15)	1.61 (1.25–2.07)
Other BBs	2.51 (2.31–2.74)	1.39 (1.26–1.52)	1.53 (1.38–1.69)

Adjusted: age, sex, eGFR, smoking, alcohol, location, family income level, Hb, BMI, albuminuria, HTN, DM, DLP, PAD, RASi, CCB, diuretics, statins, antiplatelet agents, and number of hypertensive medications.

Non-users were used as the reference group.

Abbreviations: BB, beta-blocker; CKD, chronic kidney disease; HR, hazard ratio; CI, confidence interval; PSM, propensity-score matched; MACE, major adverse cardiovascular events; CV, cardiovascular; MI, myocardial infarction; HF, heart failure.

### Sensitivity analysis

In a 1-year landmark analysis (Table S2), the associations remained directionally consistent with the main analysis. BB use remained associated with an increased risk of all-cause mortality (adjusted HR, 2.03; 95% CI, 1.88–2.19) and MACE (adjusted HR, 1.22; 95% CI, 1.15–1.30). All individual MACE components showed elevated risks, with the strongest association observed for CV death (HR, 2.17; 95% CI, 1.71–2.76), followed by non-fatal MI (HR, 1.25; 95% CI, 1.09–1.44), HF hospitalization (HR, 1.36; 95% CI, 1.23–1.49), and ischemic stroke (HR, 1.15; 95% CI, 1.06–1.25).

**Figure 3:**
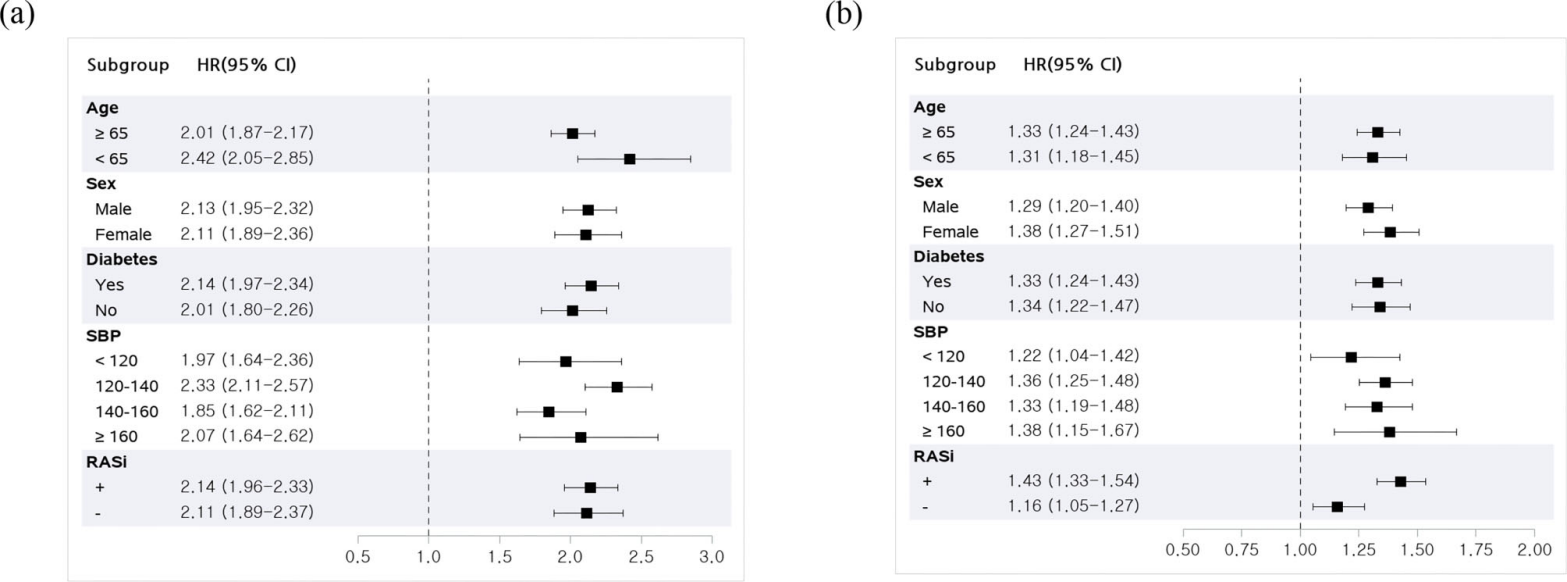
Forest plot of subgroup analysis on (a) all-cause mortality and (b) major adverse cardiovascular events.

In addition, subgroup analyses across baseline characteristics (Fig. 3) demonstrated that the associations between BB use and adverse outcomes were consistent regardless of age, sex, diabetes status, or concomitant RASi use.

## DISCUSSION

In the nationwide propensity-matched cohort of CKD patients without prior CVD, BB therapy was not associated with survival advantages and was instead linked to higher risks of all-cause mortality and major cardiovascular events. These associations appeared across CKD stages, with the pronounced excess risk observed for heart failure hospitalization. Across BB subclasses, most agents were associated with elevated mortality and MACE risks, whereas nebivolol did not increase the non-fatal MI and ischemic stroke risk. These findings suggest that routine BB therapy for primary prevention in CKD may not provide cardiovascular benefit and could be associated with adverse outcomes in certain subgroups. This observation challenges the conventional assumption that BBs are universally protective in high-risk CKD patients.

Hypertension management is pivotal to improving outcomes in CKD [27, 28]. Current guidelines recommend RASi as the first-line agents due to their renoprotective effects, but additional therapies are often needed for optimal BP control [27]. BBs have been considered theoretically attractive in CKD given the heightened sympathetic activity frequently observed in this population [29]. Although prior studies have reported survival advantages of BBs in advanced CKD patients with HF [15, 30, 31], evidence supporting their use for primary CV prevention remains limited.

Our findings contrast with reported survival benefits of BBs in CKD with HF or dialysis cohorts. Recent evidence has questioned these effects in broader or metabolically vulnerable populations. In hypertensive CKD, metoprolol was linked to higher mortality [32], and among patients with diabetes, BBs was associated with increased mortality regardless of subclass [33]. Moreover, in stable CAD patients without LV dysfunction, BB use also associated with higher risks of mortality, including CV mortality [34, 35]. Taken together, these findings suggest that BB-related benefits are context-specific and may not extend to the broader CKD population for primary CVD prevention.

BB therapy was associated with increased MACE risk, especially in HF hospitalization. This contrasts with the well-established benefits of BBs in reducing mortality in HF with reduced ejection fraction, and secondary prevention in patients after MI [36–38]. However, recent evidence suggests these benefits do not extend to populations without systolic heart dysfunction [39]. In individuals with preserved EF in U.S. registry of older outpatients, BB therapy has been associated with higher risk of HF [40]. Similarly, other CV effectiveness of BBs varies across clinical settings. Although earlier studies reported reduced mortality and recurrent events after MI [41], more recent evidence in the reperfusion era has shown neutral or diminished benefits, particularly in patients without LV dysfunction [42, 43]. For stroke prevention, meta-analysis of 13 randomized trials found that BBs are less protective than CCBs or ARBs, with a 16% higher risk of stroke [13]. Additionally, another large pooled meta-analysis reported that BB was associated with higher risks of stroke and all-cause mortality, although some benefit for uncontrolled hypertension [44]. Overall, these findings suggest that in the absence of definite CV indications, the use of BBs in CKD patients may be approached with caution.

The exact mechanisms underlying these adverse associations remain unclear. BBs improve survival in patients with systolic dysfunction by reducing heart rate and myocardial oxygen demand [45], but in individuals with preserved cardiac function or no prior CVD, such chronotropic suppression may reduce cardiac output and exercise capacity, potentially leading to adverse outcomes in CKD. In addition, impaired autonomic regulation and altered vascular reactivity in CKD may amplify the hemodynamic effects of chronic beta-blockade, resulting in suboptimal perfusion and higher ischemic or cerebrovascular risks [46]. BB therapy has also been linked to insulin resistance and new-onset diabetes, which could further exacerbate metabolic and cardiovascular risks [47]. Further mechanistic studies are warranted to clarify these pathophysiological interactions.

Given the pharmacologic heterogeneity of BBs, we performed additional subclass analyses to examine whether outcomes differed by type. Conventional BBs, such as atenolol and propranolol, are nonselective or beta1-selective agents lacking vasodilatory properties, and may reduce cardiac output and renal perfusion pressure, and adversely affect peripheral vascular and metabolic profiles in CKD [48]. Third-generation agents, nebivolol and carvedilol, possess additional vasodilatory, antioxidant, and metabolic benefits, which have shown hemodynamic benefits in patients with established systolic dysfunction [49–51]. Nevertheless, in our cohort of CKD patients without established CVD, all three BB subclasses showed similar associations with increased mortality and MACE, despite their distinct pharmacological profiles, suggesting that the cardiovascular benefits observed in established CVD populations may not extend to a primary prevention setting. Although nebivolol did not show statistically significant associations with stroke or non-fatal MI, overall risks remained elevated. These findings may partly reflect the smaller subgroup sample size, and the role of third-generation beta-blockers in CKD patients warrants further investigation.

The study has several limitations. First, as an observational study, residual confounding cannot be fully excluded. Since BBs are often prescribed to patients with higher baseline cardiovascular risk, unmeasured confounding related to detailed cardiac function or longitudinal blood pressure control may still affect the results. However, our study is based on a large, nationwide, population-based cohort, and we tried to minimize baseline differences through rigorous PS matching with extensive covariate adjustment, which substantially reduced potential confounding from pre-existing cardiovascular risk. We also incorporated time-varying adjustment for antihypertensive treatment intensity in the main model, and additional landmark analyses yielded consistent results. In addition, given the challenges of conducting randomized controlled trials in this population, our study provides meaningful real-world evidence through rigorous design and comprehensive adjustment. Second, BB exposure was defined on the basis of prescription records, which do not capture actual drug intake, dose titration, or adherence, thereby limiting the assessment of dose–response effects of beta-blocker use. Third, BBs are not pharmacologically homogeneous; differences in receptor selectivity, metabolism, and lipophilicity could lead to variable effects that were not fully addressed in this study. Although subclass analyses were conducted, more detailed evaluation across individual drug is warranted. Finally, although we applied a prespecified exposure window and conducted landmark sensitivity analyses, immortal-time bias related to exposure classification and variations in the timing of BB initiation cannot be completely excluded. In addition, because the present study did not use an active-comparator new-user design, residual confounding related to treatment selection may still persist despite extensive adjustment and PS matching.

In conclusion, BB use in patients with CKD without established cardiovascular disease was not associated with improved survival or cardiovascular outcomes, but instead showed increased risks of adverse events. However, these findings should be interpreted cautiously because residual confounding by indication, treatment selection, and immortal-time bias cannot be completely excluded. Further studies using active-comparator new-user designs or randomized trials are needed to clarify whether these associations reflect causal effects of BB therapy.

## Supplementary Material

sfag204_Supplemental_File

## Data Availability

The data that support the findings of this study are available from the Korean National Health Insurance Service (NHIS). Data are available with the permission of NHIS.
