# Supplementary material for: Higher cardiovascular risk observed with beta-blockers in CKD patients without prior cardiovascular disease
Source: Clin Kidney J. 2026 Jun 12;19(7):sfag204. doi: 10.1093/ckj/sfag204 (PMC13320235; doi:10.1093/ckj/sfag204)

**Supplementary Information**

**Contents:**

**Table S1. Variable definitions of comorbidities and outcomes using diagnostic/procedure codes**

**Table S2. Association between beta-blocker use and clinical outcomes in CKD using a 1-year landmark analysis**

**Figure S1. Kaplan-Meier curves for subclinical outcomes (MACE)**

**Table S1. Variable definitions of comorbidities and outcomes using diagnostic/procedure codes**

| **Variable** | **Definition** |
| --- | --- |
| **Medical history** |  |
| Hypertension | ICD-10 code: I10-I15 |
| Diabetes mellitus | ICD-10 code: E10-E14 |
| Dyslipidemia | ICD-10 codes: E75-78 |
| Myocardial infarction | ICD-10 codes: I21-I23 |
| Coronary intervention (IHD) | ICD-10 code: I20, I24-I25; procedure codes: O1640, O1641, O1647, O1648, O1649, OA640, OA641, OA647, OA648, OA649, M6551, M6552 |
| Peripheral vascular disease | ICD-10 codes: I70.2-3, I70.9 I73.1 I73.8-9; procedure codes: O0161-O0171, O1643-O1646 |
| Congestive heart failure | ICD-10 codes: I50 |
| Cerebrovascular disease, TIA | ICD-10 codes: I60~I69, G450, G451, G452, G454, G458, G459, G46 |
| Arrhythmia | ICD-10 code: I44-I49, R00, I48 |
| Malignancy | ICD-10 code: C00-C97 |
| COPD | ICD-10 code: J44 |
| Bradycardia | ICD-10 code: R00.1 |
| Kidney transplantation | ICD-10 code: Z94.0; procedure codes: V005 |
| Devices (Pacemaker, CRT, ICD) | Procedure/Device codes: O0203-O0207, G8201-G8205, O0211, O0212, G8301-G8103 |
|  |  |
| **Outcomes** |  |
| cardiovascular mortality | Main diagnosis in Causes of Death register (ICD-10 codes: I20-I25, I60-64) |
| Hospitalization for heart failure | ICD-10 codes: I50 |
| Non-fatal myocardial infarction | ICD-10 code: I21-I23 |
| Ischemic stroke | ICD-10 codes: I64-I64 |

ICD: International Classification of Diseases; TIA, transient ischemic attack; COPD: chronic obstructive pulmonary disease, CRT, cardiac resynchronization therapy; ICD, implantable cardioverter-defibrillator;

**Table S2. Association between beta-blocker use and clinical outcomes in CKD using a 1-year landmark analysis**

| Main Outcome |  |  | HR (95% CI) | | |
| --- | --- | --- | --- | --- | --- |
|  | No. of Events  BB/Non-BB | Incidence rate | Full cohort,  crude | Full cohort,  adjusted | PSM cohort,  adjusted |
| All-cause mortality | 1130/7177 | 15.2/8.9 | 1.71(1.61-1.82) | 1.95(1.83-2.09) | 2.03(1.88-2.19) |
| MACE | 1857/11542 | 27.2/15.0 | 1.85(1.76-1.94) | 1.20(1.14-1.27) | 1.22(1.15-1.30) |
|  |  |  |  |  |  |
| MACE components |  |  |  |  |  |
| CV death | 123/639 | 1.7/0.8 | 2.10(1.73-2.55) | 2.17(1.76-2.68) | 2.17(1.71-2.76) |
| Non-fatal MI | 365/2190 | 5.0/2.7 | 1.84(1.64-2.05) | 1.20(1.06-1.36) | 1.25(1.09-1.44) |
| Ischemic stroke | 1057/6950 | 15.0/8.9 | 1.70(1.59-1.81) | 1.14(1.06-1.23) | 1.15(1.06-1.25) |
| HF hospitalization | 826/3997 | 11.4/5.0 | 2.32(2.15-2.50) | 1.29(1.19-1.41) | 1.36(1.23-1.49) |

Adjusted: age, sex, eGFR, smoking, alcohol, location, family income level, Hb, BMI, albuminuria, HTN, DM, DLP, PAD, RASi, CCB, Diuretics, Statins, Antiplatelet agents, and number of hypertensive medications

Abbreviations: BB, beta-blocker; HR, hazard ratio; CI, confidence interval; PSM, propensity-score matched; MACE, major adverse cardiovascular events; CV, cardiovascular; MI, myocardial infarction; HF, heart failure.

**Figure Legends**

**Figure S1. Kaplan-Meier curves for subclinical outcomes (MACE)**

(a) CV death

(b) Non-fatal MI

(c) Ischemic stroke

(d) HF hospitalization

**Figure S1. Kaplan-Meier curves for subclinical outcomes (MACE)**

(a)


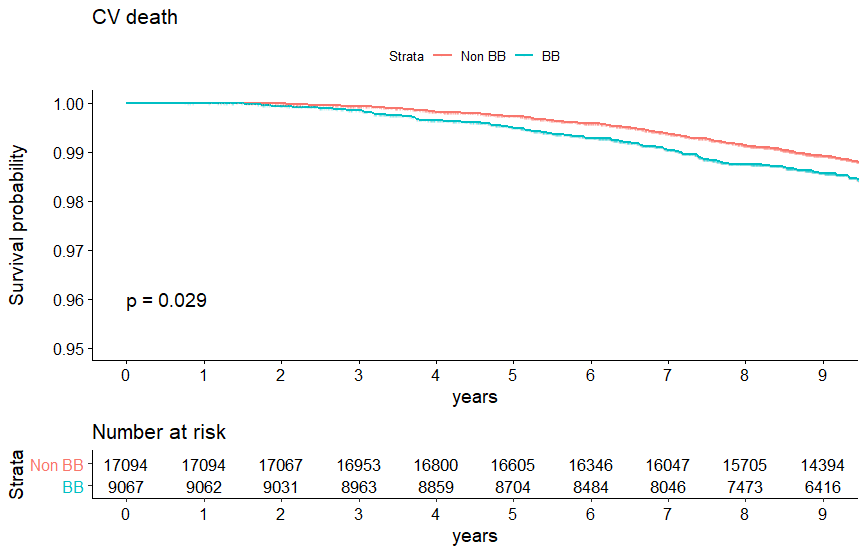


(b)


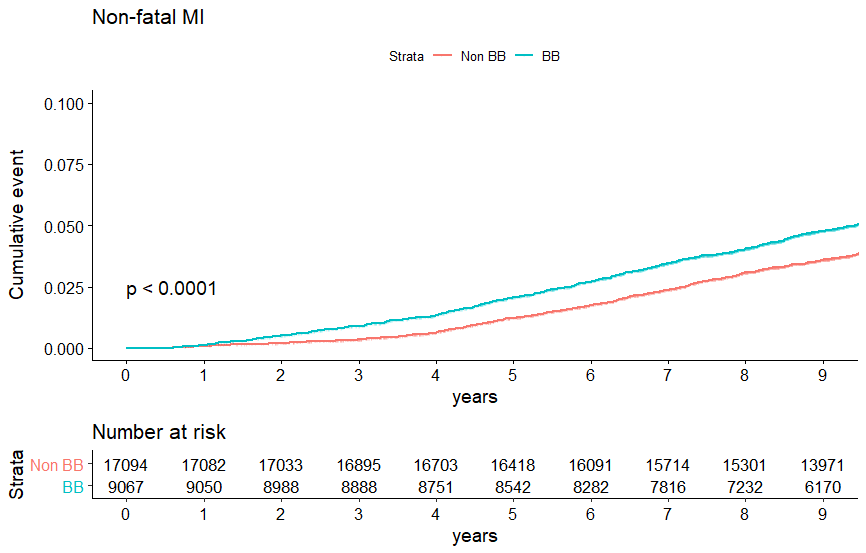


(c)


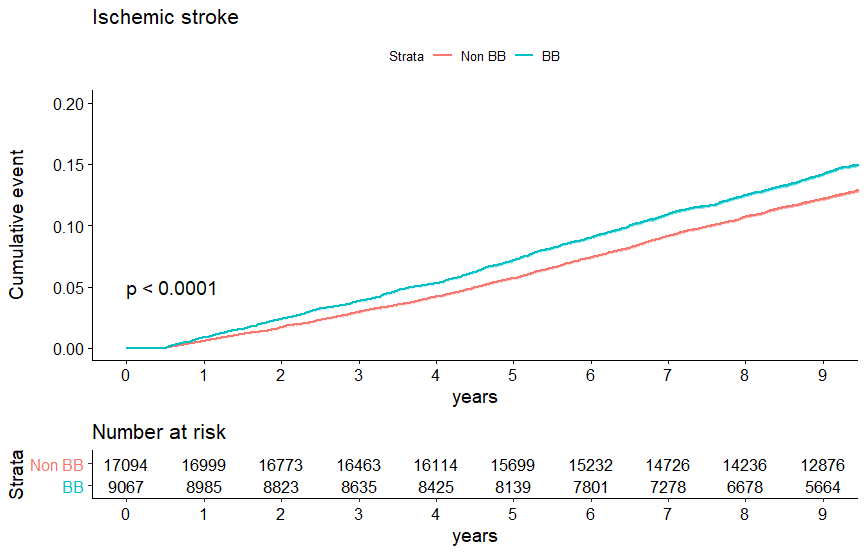


(d)


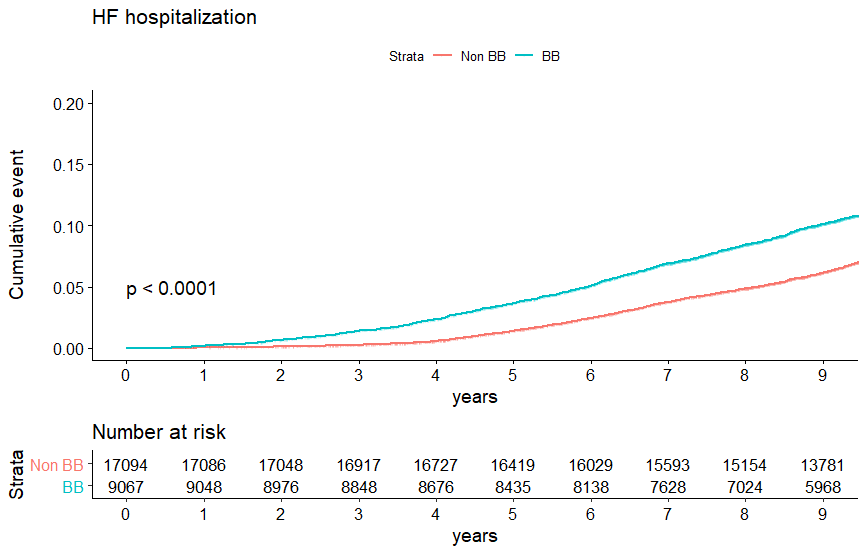

Supplement: sfag204_Supplemental_File [file sfag204_supplemental_file.docx]
